# Creating a dementia eyecare pathway for long‐term care

**DOI:** 10.1002/alz70858_101370

**Published:** 2025-12-25

**Authors:** Marianne Emma Florence Piano, Lisa Keay, Lynette Joubert, Anita M Y Goh

**Affiliations:** ^1^ National Centre for Healthy Ageing, Monash University & Peninsula Health, Melbourne, VIC, Australia; ^2^ The George Institute for Global Health, Faculty of Medicine, University of New South Wales, Sydney, NSW, Australia; ^3^ School of Optometry and Vision Science, University of New South Wales, Sydney, NSW, Australia; ^4^ Department of Social Work, University of Melbourne, Melbourne, VIC, Australia; ^5^ National Ageing Research Institute, Parkville, VIC, Australia

## Abstract

**Background:**

Seeing well matters to people living with dementia, so they can participate in activities they enjoy and maintain independence. People with dementia should be able to access person‐centred eyecare, wherever they reside. However, people with dementia are more likely to experience preventable vision loss than people without dementia, as they may miss out on regular eye tests or timely treatment for eye problems affecting vision. This risk is compounded by residing in long‐term care. To break down barriers to accessing dementia‐friendly eyecare in long‐term care facilities, this research aims to create a dementia eyecare pathway for integration into dementia care models in long‐term care.

**Methods:**

To create the pathway and an implementation plan, we will undertake research activities informed by an established implementation framework (Consolidated Framework for Implementation Research):

• Observational case study in a long‐term care facility, including observation of visits by domiciliary optometrists, and micro‐interviews with care staff, residents with and without dementia, visiting families and healthcare professionals.

• Behavioural systems mapping of case study data, to identify key actors and relationships, verified through stakeholder engagement activities.

• Audit of 25 care plans to identify how information about resident eyecare needs is documented.

• Documentary analysis of policies relating to meeting of eyecare needs in the facility, and wider policies in Australian dementia care.

• Literature review of existing domiciliary eyecare models/interventions across the world

Findings will be reviewed by an expert reference group uniting hospital, community and long‐term care perspectives on eyecare, and a project advisory group comprising people living with dementia and past/present carers. The draft pathway will be revised through two consultation rounds with the public, long‐term care sector leaders, eyecare professionals and medical specialists (e.g. geriatricians, general practitioners).

**Results:**

From these activities, final outputs will be:

1) An implementation‐ready dementia‐friendly eyecare pathway.

2) An accompanying implementation plan, developed using the implementation framework.

**Conclusion:**

Adopting a co‐design approach, together with a recognised implementation framework, allows barriers to pathway adoption/sustainment to be identified and addressed during pathway design. If everyone with dementia receives regular eye tests and eyecare, they can see well, to live well.

